# Amino acidic substitutions in the polymerase N-terminal region of a reassortant betanodavirus strain causing poor adaptation to temperature increase

**DOI:** 10.1186/s13567-019-0669-4

**Published:** 2019-06-21

**Authors:** Sandra Souto, Lucía Vázquez-Salgado, José G. Olveira, Isabel Bandín

**Affiliations:** 1 Instituto de Acuicultura, Departamento de Microbiología y Parasitología-Universidade de Santiago de Compostela, 15706, Santiago de Compostela, Spain; 2 Present Address: Unité de Virologie et d’Immunologie Moléculaires, INRA, Université Paris-Saclay, 78350 Jouy-en-Josas, France

## Abstract

Nervous necrosis virus (NNV), Genus *Betanodavirus*, is the causative agent of viral encephalopathy and retinopathy (VER), a neuropathological disease that causes fish mortalities worldwide. The NNV genome is composed of two single-stranded RNA molecules, RNA1 and RNA2, encoding the RNA polymerase and the coat protein, respectively. Betanodaviruses are classified into four genotypes: red-spotted grouper nervous necrosis virus (RGNNV), striped jack nervous necrosis virus (SJNNV), barfin flounder nervous necrosis virus (BFNNV) and tiger puffer nervous necrosis virus (TPNNV). In Southern Europe the presence of RGNNV, SJNNV and their natural reassortants (in both RNA1/RNA2 forms: RGNNV/SJNNV and SJNNV/RGNNV) has been reported. Pathology caused by these genotypes is closely linked to water temperature and the RNA1 segment encoding amino acids 1–445 has been postulated to regulate viral adaptation to temperature. Reassortants isolated from sole (RGNNV/SJNNV) show 6 substitutions in this region when compared with the RGNNV genotype (positions 41, 48, 218, 223, 238 and 289). We have demonstrated that change of these positions to those present in the RGNNV genotype cause low and delayed replication in vitro when compared with that of the wild type strain at 25 and 30 °C. The experimental infections confirmed the impact of the mutations on viral replication because at 25 °C the viral load and the mortality were significantly lower in fish infected with the mutant than in those challenged with the non-mutated virus. It was not possible to challenge fish at 30 °C because of the scarce tolerance of sole to this temperature.

## Introduction

Viral virulence is determined by multiple factors, including host cell recognition, viral replication efficiency and/or ability to deal with the host’s immune system [1]. In addition, the interaction host-virus-environment can have also relevant effects on virus production, and therefore, on virulence [2]. Regarding environmental influences, temperature has a crucial effect on viruses hosted by fish because these are ectothermic animals.

Nervous necrosis viruses (NNV), members of the genus *Betanodavirus*, are the causative agents of a serious neuropathological condition, affecting fish worldwide, known as viral encephalopathy and retinopathy (VER). The genome of betanodaviruses is composed of two single stranded positive sense RNA molecules. The RNA1 segment encodes the RNA dependent RNA polymerase (RdRp) also known as protein A, and RNA 2 codes for the coat protein [3]. In addition, a subgenomic RNA (RNA3) is transcribed from the 3′ end of RNA1 [4, 5]. Betanodaviruses have been traditionally classified, based on a small variable sequence of RNA2, into four genotypes [6]. Geographical distribution of these genotypes seems to be related to their temperature sensitivity. Thus, the red-spotted grouper nervous necrosis virus (RGNNV) genotype, which affects warm-water species, is the most widely distributed, and the one with the highest number of susceptible species; the barfin flounder nervous necrosis virus (BFNNV) genotype seems limited to cold-water fish; the tiger puffer nervous necrosis virus (TPNNV) genotype has only been described in one species in Japan, and the striped jack nervous necrosis virus (SJNNV), although for a long time it was considered limited to fish from Japanese waters, has also been detected in the Iberian Peninsula [7]. In addition, in Southern Europe, reassortants between both RGNNV and SJNNV genotypes have been isolated from Senegalese sole (*Solea senegalensis*) and other fish species [8, 9]. Temperature sensitivity of betanodaviruses seems to be regulated by RNA1 [10, 11] and more specifically by the region encoding the amino acid residues 1–445 [10]. Reassortant strains (harbouring a RGNNV-type RNA1 and a SJNNV-RNA2) isolated from Senegalese sole show 6 amino acidic changes with respect to the RGNNV genotype in this region (positions 41, 48, 218, 223, 238 and 289). In addition, whereas RGNNV strains can cause disease from 23 to 30 °C (in sea bass) to 28–30 °C (in different grouper species) [12, 13], the reassortant strain causes high mortality in sole from 18 to 22 °C [14].

This study deals with the potential association of these substitutions with a differential replicative capacity at different temperatures in vitro and in vivo. To this end, reversion to the RGNNV type of all 6 positions was accomplished by site-directed mutagenesis and the replication of mutated virus was compared with that of wild type (wt) strain in E-11 cells and in sole brain tissues. RGNNV and SJNNV strains were also used for comparative purposes in the in vitro replication studies. In addition, the effect of the mutations on virulence for sole was also analyzed.

## Materials and methods

### Viruses and cells

The betanodavirus strains used in this study were: the natural reassortant SpSsIAusc160.03 (herafter wt160) showing a RGNNV RNA1 and a SJNNV RNA2 [8], employed as the parental strain for the construction of the recombinant viruses, and two recombinant strains: r160, with a genome sequence identical to wt160 [15] and r1_445, harbouring 6 point mutations in the RNA1 and generated in this study. In addition, the RGNNV-type strain SGWak97 and the SJNNV-type strain SJNag93 were used for comparative purposes. All the strains were grown on E-11 cells using L-15 Leibovitz (Lonza, Basel, Switzerland) medium supplemented with penicillin (100 units/mL), streptomycin (100 mg/mL) and 2% foetal bovine serum (FBS, Lonza) at 25 °C. Inoculated cell cultures were checked daily for development of cytopathic effect (CPE). After destruction of cell monolayers, crude virus, i.e. cell suspensions, were collected, clarified by centrifugation at 4000 × *g* for 15 min and stored at −20 °C until used.

For virus titration, tenfold dilutions of virus were inoculated into subconfluent E-11 cells in 96-well plates (Sarstedt, Nümbrecht, Germany). The titre was calculated after incubation for 10 days at 25 °C and expressed as 50% tissue culture infective dose (TCID_50_) according to Reed and Müench [16].

Transfection experiments were performed using BSRT7/5 cells [17], kindly provided by Dr K. K. Conzelmann (Ludwig-Maximilians-Universität Munich, Germany), grown in Dulbecco’s modified Eagle’s medium (DMEM; Lonza) supplemented with 10% FBS, glutamine (2 mM/L, Lonza), penicillin (100 U/mL) and streptomycin (100 mg/mL) at 37 °C in a 5% CO_2_ humidified chamber. Geneticin (G418, 1 mg/mL final concentration) was added every two subcultures.

### Recovery of recombinant viruses

The recombinant virus termed r1_445, with amino acid substitutions at position 41, 48, 218, 223, 238 and 289 in the polymerase protein, was generated by reverse genetics as described previously [15]. A cDNA clone containing the complete sequence of the wt160 (pBS160R1) [15] was used as template to introduce point mutations through site-directed mutagenesis (QuikChange site-directed mutagenesis kit; Stratagene, La Jolla, CA, USA) using the primers listed in Table 1, obtaining pBS160R1_1_445_. BSRT7/5 monolayers were transfected with a mixture of point mutated pBS160R1_1_445_ (1 µg) and pBS160R2 (1 µg) [15] by using the Lipofectamine2000 reagent (Invitrogen, Waltham, MA USA) according to the supplier’s instructions. Cells were incubated for 24 h at 37 °C and then shifted to 28 °C for 7 days. Cells were suspended in the supernatant by scraping the wells and then subjected to freezing/thawing and clarification by centrifugation. Supernatants were subjected to several passages on E-11 cells until CPE was observed. Virus progeny integrity was confirmed by sequencing the region of the RNA1 which contains the desired substitutions (GATC Biotech, Ebersberg, Germany) amplified using the commercial kit Go Taq Flexi DNA Polymerase (Promega, Madison, WI, USA) and the primers NNVs1_1f and NNVs1_2r [8]. Sequencing was also used after each experiment to confirm the identity of viral recombinants.

**Table 1:** **Oligonucleotides used for mutagenesis and positions of the point mutations in strain r1_445**

Oligonucleotide sequence (5′ to 3′)^a^	nt substitution^b^	aa substitution
AGGATTATCGCCAACGCGTCATCGCTGAGAAGAAA	A-199 → G	Iso-41 → Val
CGCGTCATCGCTGAGAAGAAACAAATTCTCCGTGATGC	G-220 → A	Val-48 → Iso
CCCGACCTCGAGGTTTCTGGGCGAATC	C-730 → T	Leu-218 → Phe
GAGGTTTCTGGGCGAATCTGATGCAGATCCTG	T-745 → A	Leu-223 → Met
GTCACCGCGATCTGTAGTTTTCTTTACACCAAGC	A-791 → T	Thr-238 → Phe
CAAGATCAGTGAGTATGGTGCTGAGTTGGAGTATATGCG	A-943 → G	Thr-289 → Ala

^a^Nucleotides used for mutagenesis are underlined.

^b^Positions in SpSsIAusc160.03 RNA1 genome (GenBank accession no. NC_024492.1).

### Effect of temperature on viral replication in vitro

E-11 cells grown in 25 cm^2^ flasks were infected with either wt160, r160, r1_445, SGWak97, or SJNag93 (4 flasks/strain) at a multiplicity of infection (MOI) of 0.01. The negative control was inoculated with L-15 medium. After 1 h adsorption at 15, 20, 25 or 30 °C, the cells were washed three times with PBS and then L-15 with 2% FBS was added to each flask which were incubated at the temperature used for adsorption. Infected cultures were checked daily for CPE appearance up to 10 days. Every day an aliquot of 700 μL of supernatant was sampled and subsequently replaced with the same volume of fresh medium. These samples were subjected to viral titration and RT-qPCR.

### Effect of high temperature on virus adsorption

In order to assess the effect of high temperature on viral adsorption to E-11 cells, infectivity capacity was evaluated at 25 and 30 °C. Each virus was inoculated at a MOI of 0.01 in 48-well plates. After an adsorption period (1 h), the remaining inoculum was removed and maintained at −20 °C until RNA1 quantification by RT-qPCR. Two quantification sets were performed: the original inoculum and the remaining inoculum from each of the inoculated wells (non-adsorbed virus), used to calculate the adsorbed virus.

### Effect of temperature on viral replication in vivo and virulence

Senegalese sole (*S. senegalensis*) juveniles (mean weight 1.5 g) were obtained from a commercial fish farm and maintained at the fish facilities of the Universidade de Santiago de Compostela in opaque tanks containing seawater. Prior to experimental infection sole were acclimated at each experimental temperature for a minimum of 10 days. During this period 10 fish were sacrificed with an anesthetic overdose (MS-222, Sigma-Aldrich, St. Louis, MO, USA) and used for the diagnosis of bacterial pathogens as well as for four regular viral agents: infectious pancreatic necrosis virus (IPNV), infectious hematopoietic necrosis virus (IHNV), viral haemorrhagic septicaemia virus (VHSV) and betanodavirus. Bacterial isolation was accomplished by inoculating samples of kidney, spleen, and liver onto tryptone soy agar supplemented with 1% (wt/vol) NaCl (TSA-1) and TCBS (thiosulfate-citrate-bile salts-sucrose) agar (Vibrio selective agar) and incubating the samples at 25 °C for 24 h. Viral detection was performed by RT-PCR as previously described [18]. Fish were fed *at libitum* once a day and handled in strict accordance with good animal practices as defined by the European Union guidelines for the handling of laboratory animals (directive 2010/63/UE). During the experiment oxygen, nitrogen compounds, pH, and salinity were monitored continuously. Temperature, lighting and noise were also strictly controlled in order to minimize stress.

Sole juveniles were infected with the recombinant (r160 and r1_445) at 15, 20 and 25 °C. Triplicate sole groups (*n* = 20/temperature assayed) were bath infected with each strain at a virus concentration of 10^5^ TCID_50_/mL for 3 h with strong aeration. Control fish were handled like the infected groups and L-15 medium was used for mock infection. Mortalities and clinical signs were recorded daily, and dead fish were removed. Surviving fish were euthanized using a MS-222 overdose. Brain tissues from both dead fish and survivors were aseptically collected and pooled in samples comprising tissues from 3 fish. Samples were analyzed by cell culture and RNA quantification.

### Processing of samples

Brain tissues were analyzed in pools of three, except when the number of available fish was too low, in which case they were processed individually. In both cases samples were homogenized in Earle’s balanced salt solution (1:10 for individual brains and 1:5 for pooled samples) supplemented with antibiotics (1000 IU/mL penicillin, 1000 µg/mL streptomycin, 500 µg/mL gentamycin and 500 µg/mL partricin). The samples were centrifuged at 3000 × *g* for 15 min at 4 °C. The resulting supernatants were split into two aliquots; one stored at −80 °C for later use in RT-qPCR, and the other used for inoculation onto monolayers of E-11 cells in final dilutions of 1:100 and 1:1000. The plates were incubated at 25 °C and monitored for cytopathic effect (CPE) for 7 days.

### RNA quantification

Total RNA was extracted from infected cell culture supernatants and tissue homogenates using the EZNA Total RNA I kit (Omega Biotek, Norcross, GA, USA) following manufacturer’s instructions. Extracted RNA was reverse transcribed with the Superscript IV reverse transcriptase (Invitrogen) using random primers at 50 °C for 50 min, followed by 5 min at 85 °C for RT enzyme inactivation. For qPCR, reactions were processed with 2 µL of cDNA samples in 20 µL final volume, using iQTM SYBR^®^Green Supermix (Bio-Rad, Hercules, CA, USA) and 200 nM of primers SnodR1 F/R [18]. Reactions were carried out in a CFX96TM Real-Time PCR Detection System (Bio-Rad) as previously described [15], briefly after a denaturation/activation step at 95 °C for 15 min, the mixture was subjected to 40 cycles of amplification (denaturation at 95 °C for 15 s, annealing and extension at 60 °C for 15 s). All samples were tested in triplicate. Quantification of genome copies was accomplished using standard curves generated from 20-fold serial dilutions of a plasmid DNA containing the full-length RNA1 of strain SpSs-IAusc160.03 in the range of 10^1^ to 10^7^ copies/µL. Viral load data were calculated as RNA1 copies per g of fish tissue (tissue homogenates) or per mL of supernatant (cell culture).

### Statistical analysis

Statistical analyses were carried out using GraphPad Prism version 7.00 for Windows (GraphPad Software, La Jolla, CA, USA). Viral quantification (TCID_50_ and RNA1 copies) data were subjected to a two-way ANOVA followed by Tukey’s multiple comparisons. Mortality rates were analysed by the survival curves, using the Kaplan–Meyer test. To determine significant differences in survival distributions between experimental groups, a log-rank Mantel Cox test was carried out. *p* < 0.05 was considered statistically significant.

## Results

### Recovery of the mutant strain r1_445

Site-directed mutagenesis was used to perform 6 nucleotide changes in the RNA1 genome of r160 (one nucleotide change per amino acid substitution in the RdRp sequence) which removed the differences observed between the wt160 sequence and the sequence of SGWak97 (GenBank accession number NC_008040). Resultant amino acid substitutions affected to the following positions in the protein A sequence: 41 (Ile → Val), 48 (Val → Ile), 218 (Leu → Phe), 223 (Leu → Lys), 238 (Tyr → Phe) and 289 (Thr → Ala). Presence of mutations was confirmed by sequencing. CPE was observed in the first passage on E-11 cells, and then r1_445 was amplified by two successive passages. Viral titre at passage 3 reached 10^8^ TCID_50_/mL.

### Effect of temperature on viral replication in vitro

Inoculated flasks were visualized/checked daily for the appearance of CPE (Table 2). At 15 °C, no specific alterations associated to betanodavirus infection (i.e. vacuolization of cells) were observed. Cell shrinkage was observed in all flasks probably because of incubation at a non-optimal temperature. After 3 days post-infection (dpi) cells infected with RGNNV strain appeared contracted and started to detach from the flask surface. At 6 and 8 dpi, cells inoculated with SJNNV and wt160 strains and those infected with r160 strain, respectively, showed similar characteristics. However, complete disruption of the cell monolayer was not observed in any of the flasks. No cell detachment was observed in the flask infected with r1_445. At 20 °C, typical betanodavirus CPE was observed after 5–6 days which led to the complete destruction of the monolayers, except the cells infected with r1_445. At 25 °C all strains developed CPE after 3 dpi and the monolayer was destroyed in all cases; but this disruption was delayed 2 days in wells inoculated with r1_445, with respect to those inoculated with r160 and wt160. At the highest temperature tested, 30 °C, CPE was also observed after 3 days in most of the flasks, and complete destruction at 5 dpi. As occurred at 25 °C, total monolayer disruption was delayed 2 days (until day 7) in the flasks infected with r1_445. No destruction was accomplished in flasks infected with the SJNNV strain (Table 2).

**Table 2:** **Cytopathic effect (CPE) observed in E-11 cells inoculated with the mutated virus r1_445, the 160 strain (wt and r) and parental strains from genotypes RGNNV and SJNNV at different temperatures**

Days pi	Temperature (°C)																			
	15					20					25					30				
	RGNNV	SJNNV	wt160	r160	r1_445	RGNNV	SJNNV	wt160	r160	r1_445	RGNNV	SJNNV	wt160	r160	r1_445	RGNNV	SJNNV	wt160	r160	r1_445
1	S	S	S	S	S	−	−	−	−	−	−	−	−	−	−	−	−	−	−	−
2	S	S	S	S	S	−	−	−	−	−	−	−	−	−	−	+	−	−	−	−
3	S, D	S	S	S	S	−	−	−	−	−	+	+	+	+	+	++	+	+	++	+
4	S, D	S	S	S	S	−	−	−	−	−	++	+	+	+	+	+++	+	++	+++	+
5	S, D	S	S	S	S	+	+	+	−	−	+++	+++	++	++	++	++++	+	++++	++++	+++
6	S, D	S, D	S, D	S	S	++	++	+	+	+	++++	++++	+++	+++	++	NA	+	NA	NA	+++
7	S, D	S, D	S, D	S	S	++	++	++	++	+	NA	NA	++++	++++	+++	NA	+	NA	NA	++++
8	S, D	S, D	S, D	S, D	S	+++	+++	+++	+++	+	NA	NA	NA	NA	+++	NA	++	NA	NA	NA
9	S, D	S, D	S, D	S, D	S	++++	++++	+++	++++	++	NA	NA	NA	NA	++++	NA	+++	NA	NA	NA
10	S, D	S, D	S, D	S, D	S	NA	NA	++++	NA	+++	NA	NA	NA	NA	NA	NA	+++	NA	NA	NA

S: Cell shrinkage, D: detached cells, − absence of cytopathic effect (CPE), + initial vacuolation, ++ extensive vacuolation, +++ extensive vacuolation and initial disruption of the monolayer, ++++ complete destruction of the monolayer, NA: not applicable.

**Figure 1:**
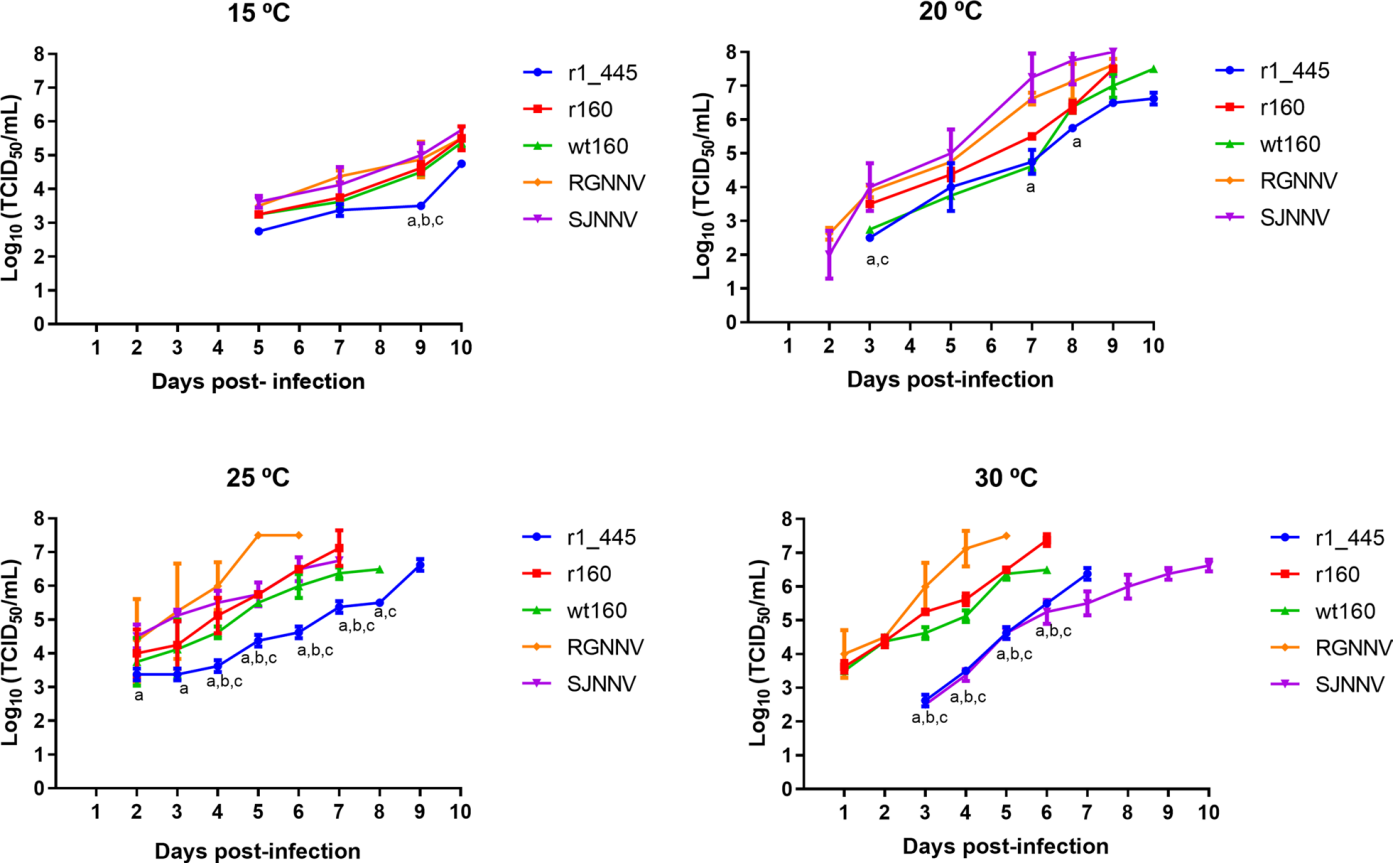
**Viral replication on E-11 cells.** Data are expressed as TCID_50_/mL. Means and standard deviations from three wells are presented. Letters indicate significant differences (*p* < 0.05) with: a RGNNV; b wt160; c r160.

**Figure 2:**
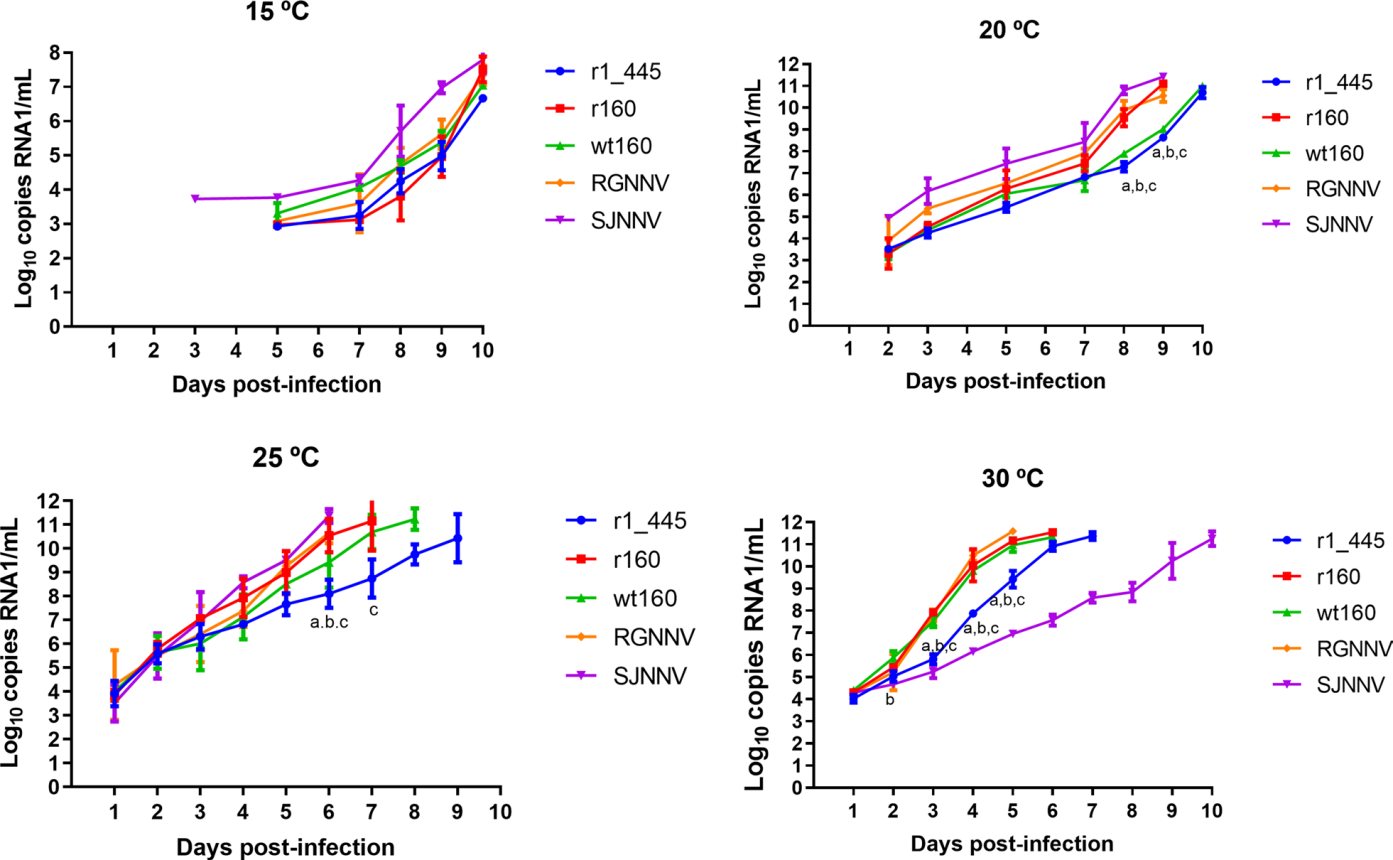
**Viral replication on E-11 cells.** Data are expressed as viral RNA1 detected in E-11 cells supernatants. Means and standard deviations from three wells are presented. Letters indicate significant differences (*p* < 0.05) with: a RGNNV; b wt160; c r160.

Production of infectious virus was assessed by TCID_50_ method (Figure 1). At 15 °C low viral titers were obtained and only from day 5 onwards. At 5 dpi the lowest value was displayed by the mutant strain r1_445 (mean titre 5.6 × 10^2^ TCID_50_/mL) and the highest one by SJNNV strain (4.2 × 10^3^). At 10 dpi the lowest and highest titers were also obtained with r1_445 and SJNNV strains (5.6 × 10^4^ and 7.5 × 10^5^ TCID_50_/mL, respectively). Even though r1_445 production was significantly lower than the other strains only at day 9, differences around 1 log were observed between the mutant r1_445 and the non-mutated recombinant r160 throughout the whole experiment. Incubation at 20 °C provided more efficient replication results and titer values were recorded from 2 to 3 dpi. As occurred at 15 °C, the mutant r1_445 displayed the lowest titer value at 3 dpi (3.2 × 10^2^ TCID_50_/mL) which was 1 log lower than that of the non-mutated recombinant strain r160 (3.2 × 10^3^), although it was quite similar to the wt160. However, from day 3 onwards significant differences were observed only with RGNNV strain. At 25 °C all five strains tested showed increased replication values. However, as recorded at lower temperatures, r1_445 showed slower replication than the other strains. The mutant strain r1_445 showed significant lower production (*p* < 0.05) comparing with RGNNV strain from 2 dpi, with r160 from 4 to 8 dpi and with wt160 from 4 to 7 dpi. At day 7 pi whereas r160 showed a maximum titer value of 1.3 × 10^7^ TCID_50_/mL, the titer displayed by r1_445 was 2 logs lower (1 × 10^5^), reaching the maximum titer of 4.2 × 10^6^ 2 days later. Finally, at 30 °C titer was obtained as early as 1 dpi from RGNNV, wt160 and r160 strains (3.2 to 4.3 × 10^4^ TCID_50_/mL). These values, increased to 4.2 × 10^4^–1 × 10^6^ TCID_50_/mL at 3 dpi when first titers were obtained from SJNNV and r1_445 (2.4 and 3.2 × 10^2^, respectively). From this time point to 6 dpi significant differences (*p* < 0.0001) were observed between r1_445 and either, r160, wt160 and RGNNV. RNA1 quantification supported the results obtained by titration, although differences among strains were slightly lower (Figure 2). At 15 °C SJNNV strain showed the highest values, being the only one detected at 3 dpi (mean RNA copy number 5.3 × 10^3^/mL) and reaching the maximum value of 1.1 × 10^7^ RNA copies/mL at 10 dpi. The other four strains were detected 2 days later (5 dpi) with similar values (8.5 × 10^2^–2.3 × 10^3^). Although significant differences were not observed among the strains, the genomic load obtained from r1_445 at 10 dpi was 1 and 0.7 log lower than that of r160 and wt160, respectively. As it can be observed in Figure 2 at 20 °C all five strains were detected at 2 dpi and SJNNV strain showed again the highest RNA copy number. During the first 3 days the genomic load of r1_445, r160 and wt160 was very similar. However, from day 5 pi onwards whereas the values of r1_445 and wt160 remained very close, comparison of r1_445 with r160 replication showed clear differences. At 8–9 dpi these differences (2–3 logs) were significant (*p* < 0.0001). As growth temperature increased viral genomes were detected from the first day (25 and 30 °C). At 25 °C RGNNV strain showed the highest initial values, although at 5 dpi SJNNV and RGNNV strains showed almost identical genomic load. As occurred at lower temperatures the mutant r1_445 showed a delay in the replication. At 6 dpi significant differences (*p* < 0.05) were observed between r1_445 and wt160, r160 and RGNNV strains. At 7 dpi when r160 reached the maximum copy number (5.4 × 10^11^), the value obtained from r1_445 was 2 logs lower. The mutant strain reached a final genomic load of 7.4 × 10^10^, at 9 dpi. Finally, at 30 °C, the five strains showed similar initial and final genomic loads (1.1–2.5 × 10^4^ and 2.1–4.5 × 10^11^, respectively). However, differences in the time needed to reach the final value were clear and although the RGNNV strain showed the highest genomic load at 5 dpi, SJNNV strain did not reach the peak until 10 dpi. Regarding the mutant r 1_445 between 3 and 5 dpi it showed a delay in replication with respect to both r160 and wt160 with a difference in the genomic load of 2–3 logs (*p* < 0.0001). The delay was maintained until the end of the experiment, because although no significant differences were observed in the final genomic load obtained from the three strains, r1_445 took 1 day more than r160 and wt160 to reach the maximum level.

### Effect of increased temperature on viral adsorption

The adsorbed virus was estimated from the difference between the original viral inoculum and the non-adsorbed virus using RNA1 quantification values. The efficiency of the adsorption was then calculated from the ratio between adsorbed virus and total viral inoculum. At 25 °C all strains showed adsorption rates above 99%. At 30 °C although no significant differences (*p* > 0.99) were found between strains r1_445 and SJNNV showed slightly lower values (Table 3).

**Table 3:** **Viral attachment to E-11 cells at 25** **°C**

Viral strain	Inoculum^3^		Adsorbed virus^4^					
			25 °C			30 °C		
	RNA1 copies^1^	Log ± SD^2^	RNA1 copies	Log ± SD	Ad rate^5^	RNA1 copies	Log ± SD	Ad rate
wt160	4.10 × 10^11^	11.61 ± 0.14	4.08 × 10^11^	11.61 ± 0.31	99.51	4.07 × 10^11^	11.61 ± 0.31	99.26
r160	7.24 × 10^10^	10.86 ± 0.15	7.19 × 10^10^	10.86 ± 0.15	99.31	7.17 × 10^10^	10.85 ± 0.15	99.03
r1_445	3.22 × 10^11^	11.51 ± 0.25	3.19 × 10^11^	11.50 ± 0.24	99.07	3.18 × 10^11^	11.50 ± 0.24	98.75
RGNNV	5.62 × 10^10^	10.75 ± 0.18	5.60 × 10^10^	10.75 ± 0.08	99.64	5.58 × 10^10^	10.75 ± 0.08	99.28
SJNNV	1.71 × 10^11^	12.23 ± 0.15	1.70 × 10^11^	11.23 ± 0. 27	99.41	1.69 × 10^11^	11.23 ± 0.15	98.83

Results expressed as RNA1 copies/mL (1) and log_10_RNA1 copies/mL ± standard deviation (2) from 3 replicas of inoculated (3) and adsorbed virus (estimated as the difference between the RNA1 values of the original inocula and those of the remaining inocula after 1 h adsorption) (4) The efficiency of the adsorption (5; as percentage) was calculated from the ratio between adsorbed and total viral inoculum.

### Effect of temperature on viral replication in vivo

**Figure 3:**
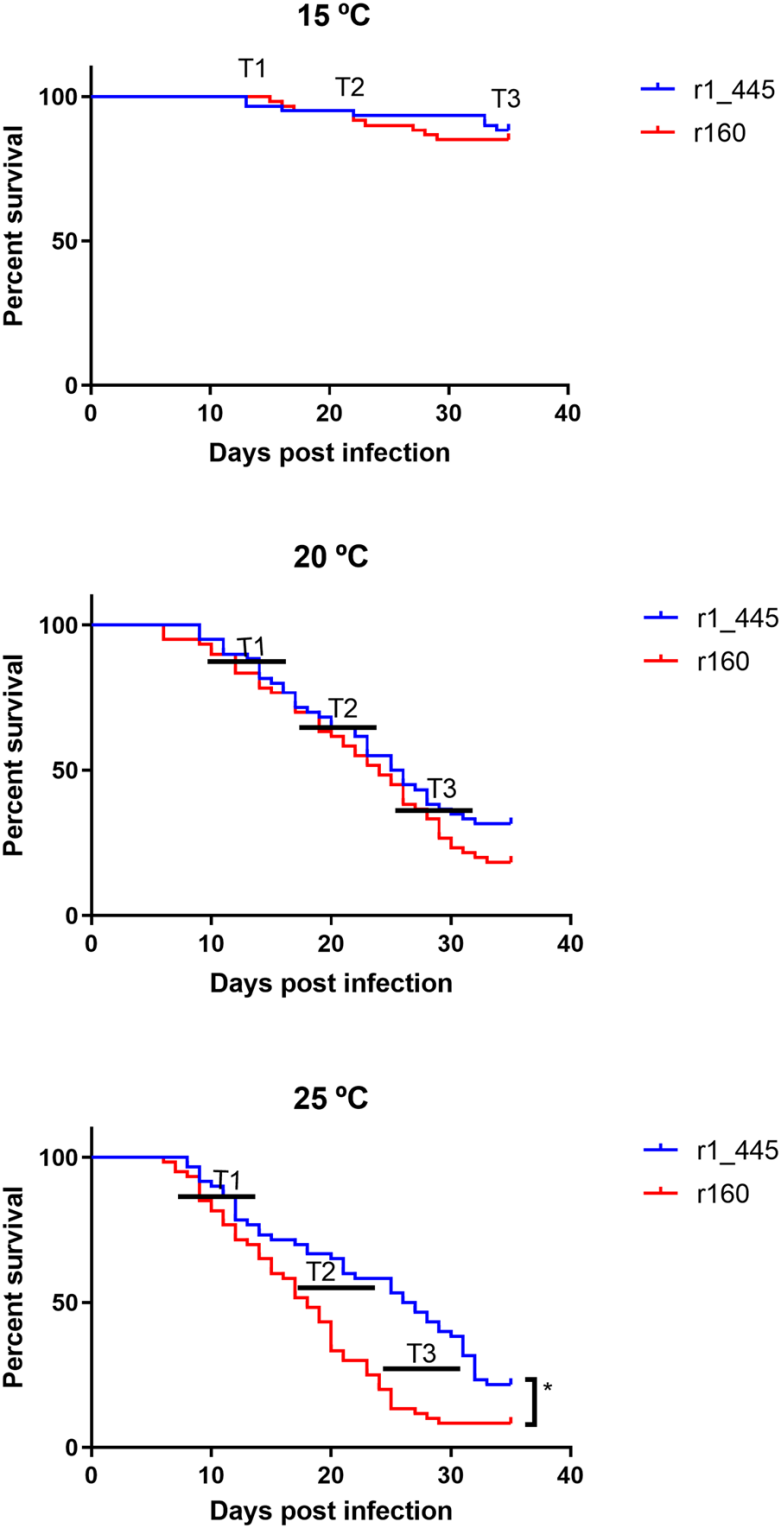
**Virulence of viral strains for Senegalese sole.** The curves represent the fish survival after infection by immersion with r160 and the mutant r1_445 at 15, 20 and 25 °C. Fish were infected by bath at a final concentration of 10^5^ TCID_50_/mL. Values are expressed as mean ± SD (*n* = 3). Asterisk indicates significant differences (*p* < 0.05).

Experimental challenges were performed only with recombinant r160 and mutant r1_445 strains in order to minimize the number of fish and because r160 and w160 has been previously demonstrated to cause similar mortalities [15]. Fish infections were performed at 15, 20 and 25 °C. High mortalities were recorded at 20 and 25 °C, whereas at 15 °C survival rates were around 85% for both strains (Figure 3). At 20 and 25 °C, the number of survivors was higher in the tanks challenged with r1_445 than in those infected with r160 (31.7 versus 18.3% and 49.7 versus 14.1%, at 20 and 25 °C, respectively), although significant differences were observed only at 25 °C (*p* < 0.001); at this temperature mortalities in fish infected with r1_445 were clearly delayed with respect to those observed in fish infected with r160.

**Figure 4:**
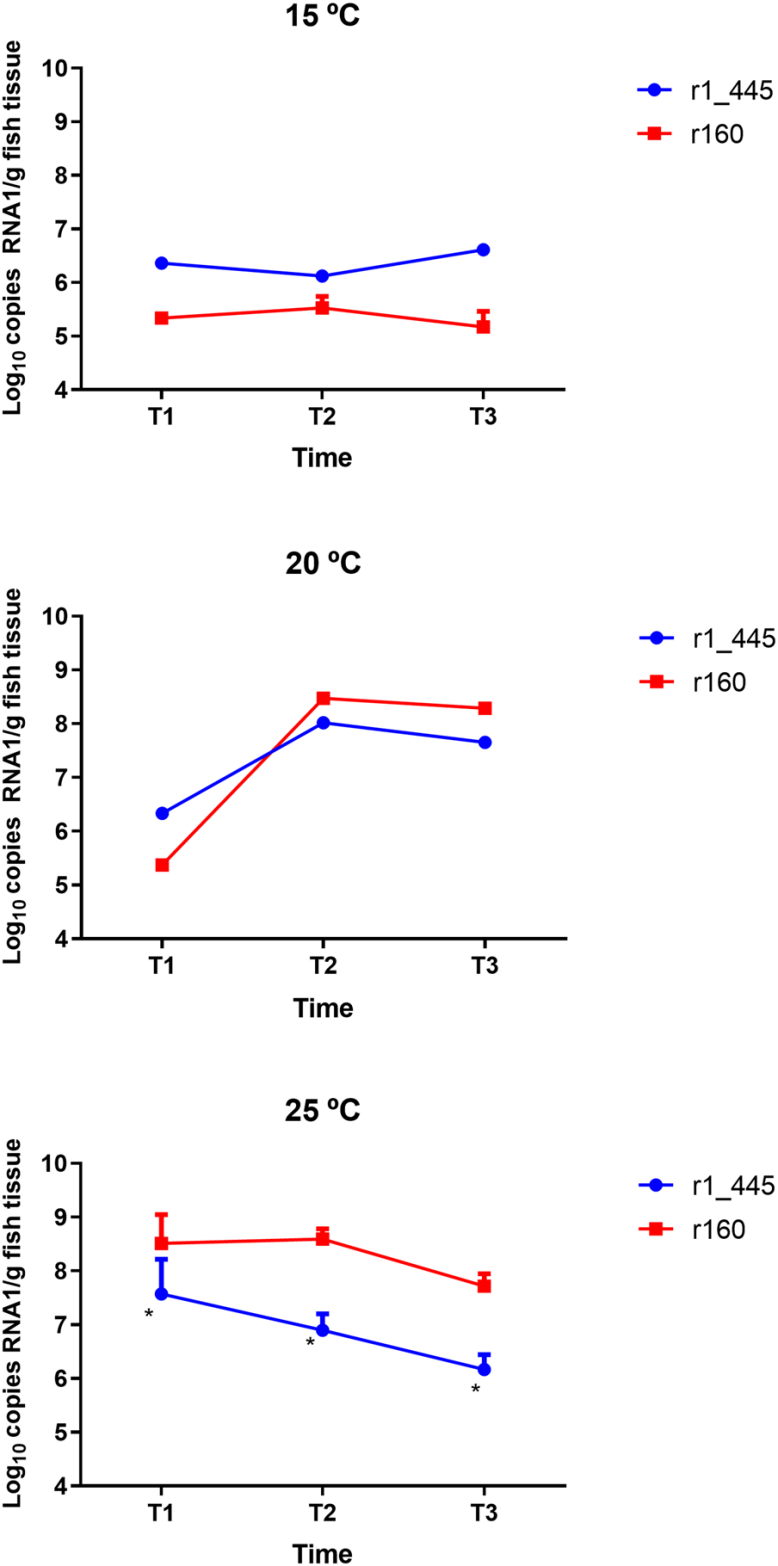
**Viral replication in sole brain tissues infected with r160 and r1_445 maintained at 15, 20 and 25** **°C.** Numbers on the y axis represent the number of RNA1 copies per gram of brain tissues and the numbers on the x axis indicate the different mortality phases T1, initial mortality phase; T2, half-stabilized phase; T3, late mortality phase. Asterisk indicates significant differences (*p* < 0.05) with r160.

Infective particles were recovered in cell culture from all the dead fish samples regardless of the temperature used in the challenge. To analyze the RNA1 loads in infected fish three different phases in the mortality curve were considered and named as: (i) T1, initial mortality phase, (ii) T2, acute mortality phase and (iii) T3, late mortality phase (Figure 4). Depending on the phase and temperature fish were analyzed individually or in pools of three. Differences in the RNA1 copy number were observed at the different temperatures tested and between both strains. At 15 °C, although the viral genomic load was low in all samples, fish infected with r1_445 showed values around 1 log higher than those infected with r160 (10^6^ and 10^5^ mean RNA copy number/g brain tissue, respectively). As challenge temperature was higher viral loads obtained from infected fish also increased. At 20 °C fish infected with r1_445 showed a higher average RNA1 load than those challenged with r160 (2.1 × 10^6^ and 2.1 × 10^5^, respectively) only at T1. However, at T2 and T3 the opposite situation was observed and RNA1 copy number obtained from fish infected with r160 (2.3 and 1.9 × 10^8^) was slightly higher than that of r1_445-infected fish (9.7 and 4.5 × 10^7^). At 25 °C viral genomic load showed by fish infected with r160 was significantly higher than that obtained from fish challenged with r1_445 (1–1.5 log) at all three times. At T1 and T2 fish infected with r160 showed similar values (5.6 and 4.3 × 10^8^) clearly higher than those observed in groups challenged with the mutant strain (6.0 × 10^7^ and 9.1 × 10^6^). Although, a decrease of around 1 log was observed in the last mortality phase (T3) regardless the strain analyzed, virus challenged with the non-mutated virus showed a significant higher viral load (*p* < 0.01). The viral load obtained from surviving fish was slightly lower than that achieved with dead fish at the three temperatures. At 15 and 20 °C survivors infected with both strains showed similar viral load (mean copy number 2.2–5.2 × 10^4^ and 7.2–9.6 × 10^5^, at 15 and 20 °C, respectively). At 25 °C as occurred with dead fish, differences between both strains were higher (6.6 × 10^6^ and 7.3 × 10^5^, survivors infected with r160 and r1_445, respectively).

## Discussion

Two of the four betanodavirus genotypes, RGNNV and SJNNV, as well as their reassortants (either RGNNV/SJNNV or SJNNV/RGNNV) are considered pathogenic for warm-water species [19]. Temperature increase plays an important role in the induction of the disease caused by these genotypes and it is considered a predisposing factor for disease outbreaks [12]. Previous studies have indicated that RNA1 plays a key role in controlling viral replication at different temperatures [10, 11] and that the region comprising 1–445 amino acid positions of the protein A is involved in NNV thermotolerance [10]. The reassortant strains (RGNNV/SJNNV) isolated from Senegalese sole in the Iberian Peninsula show 6 amino acid changes with respect to the RGNNV genotype in this region [8]. With the aim to analyze the role of the above cited positions in the viral adaptation to temperature, the replication of a recombinant virus showing point mutations has been compared with that of the recombinant with no mutations, the wild type strain and strains belonging to the parental genotypes RGNNV and SJNNV at 15, 20, 25 and 30 °C. In addition, the effect of the mutations on the virulence for sole has been analyzed.

The replication assays performed in vitro showed that all strains replicate at the four temperatures tested as it has been previously reported [11]. However, at 15 °C the lowest viral production was obtained, indicating that this is a suboptimal temperature for all 5 strains analyzed. As temperature increased viral yields were higher but whereas at 20 °C only slight differences were observed between the mutant r1_445 and 160 (both wt and recombinant), the effect of mutations was obvious at 25 and 30 °C. At both temperatures the replication of r1_445 was significantly lower (*p* < 0.05) than that of wt160 and delayed in time at 30 °C. The analysis of the viral load supports these results. These findings indicate that r1_445 shows a temperature sensitivity (*ts*) phenotype and confirm that 1–445 region of RNA polymerase is involved in the temperature adaptation of NNV as previously reported [10]. This region has transmembrane domains (TMD) which can function as membrane localization signals (MLS) [20, 21]. These MLS mediate protein A localization within the mitochondria membrane where the replication complex is formed [20]. Three putative TMD located at positions 6–26, 152–173 and 224–249 have been identified in the four NNV genotypes and 9 amino acid signatures characteristic for each genotype were recognized at positions 7, 19, 155, 223, 232, 235, 241, 251 and 254, which could be related with differences in growth kinetics among genotypes [11]. Position 223 is one of the 6 substitutions observed in the reassortant RdRp and that has been mutated in r1_445 to the residue present in the RGNNV type. Substitution at this position (Leu → Lys) implies an important change in the physico-chemical properties (hydrophobic side change → positively charged chain). Although aa 223 is flanking the 224–249 TMD, it has been suggested that the net positive charge in the flanking regions of the TMD plays most important role than the TMD sequence itself [22]. Other important change could be that of position 238 (Tyr → Phe) which is within the aforementioned TMD, but in this case the substitution does not seem to have drastic effects. Therefore, the low tolerance to high temperatures observed in the mutant r1_445 seems to point to position 223 as a putative responsible for temperature regulation, at least in the reassortant strains, and suggest that the *ts* could be related to difficulties in the mitochondrial recognition. However, it is interesting to note that although point mutations shown by the mutant strain made the 1–445 region identical to that of the RGNNV strain, as temperature increases, r1_445 replication was clearly slower than that of this isolate. This finding suggests that temperature sensitivity might be controlled not only by the N-terminal side of RNA1, but also by different regions as in other RNA viruses, like flaviviruses [23, 24] or paramyxoviruses [25, 26], in which *ts* phenotype was conferred by a number of mutations throughout structural and non-structural genes. The natural reassortant strain shows other differences with the parental genotypes, in both RNA1 and RNA2. In RNA1 these differences consist of 13 additional amino acid positions in the RdRp coding region, [8] and four nucleotides in the 3′ NCR [15]. In RNA2 the mismatches with the SJNNV genotype consist of five to six amino acids in the capsid protein, three shared by all strains [8], and five nucleotides in the 3′NCR [15]. RNA2 substitutions have been demonstrated to play a role in virulence for fish [15, 27–29]. Substitutions in protein A other than in the 1–445 region could be a consequence of the complex phenomenon of reassortment but could also be involved in virulence or thermotolerance. Generation of recombinants harbouring mutations in the 1–445 region and in different positions of RNA1 are in progress to elucidate this issue.

The experimental infections confirmed the impact of the mutations on the viral replication because at 25 °C the viral load was significantly lower in fish infected with the mutant virus than in those challenged with the non-mutated virus. The *ts* phenotype is associated with a decreased viral replication and attenuation at a restrictive temperature in different viruses [23, 25, 30–32]. Although at 25 °C the mutant r1_445 caused significantly fewer mortalities which were delayed in time with respect to those caused by the non-mutated strain a complete attenuation was not achieved. Unfortunately, it was not possible to challenge fish at 30 °C because of the scarce tolerance of sole to temperatures above 25 °C [33] and thus the effect of this increased temperature on viral virulence remains unknown.

One of the steps of the viral cycle that could be affected by temperature increase is the attachment to host cell surfaces. However, the adsorption capacity of the mutant r1_445 to E-11 cells was not affected by high temperatures (25 and 30 °C). This result agrees with a previous study showing similar level of binding of the four NNV genotypes at the aforementioned temperatures [10]. The interaction between NNV and host cells is produced through the P-domain of the capsid protein [34] and the wt strain shows two amino acid substitutions in this region which have been demonstrated to affect the interaction with sole neural cells [35]. Further studies will analyze if this interaction is affected by increasing temperature.

Results of this study clearly demonstrated that modification of amino acids in region 1–445 of a reassortant betanodavirus RdRp can affect the outcome of viral infection at high temperatures. This poor adaptation to high temperatures might be related to difficulties in the formation of the complex replication. However, although the amino acid positions of the reassortant were changed to RGNNV-type, mutant r1_445 did show significantly lower replication than RGNNV strain at 25 and 30 °C, suggesting that genomic regions other than 1–445 may be involved in NNV thermotolerance. The identification of these genomic determinants could lead to the obtention of a complete attenuated virus that in turn could be used in vaccine formulations for preventing VER outbreaks.

## Data Availability

The datasets used and/or analysed during the current study are available from the corresponding author on reasonable request.

## Abbreviations

NNV: nervous necrosis virus
SJNNV: striped jack nervous necrosis virus
RGNNV: red-spotted grouper nervous necrosis virus
TPNNV: tiger puffer nervous necrosis virus
BFNNV: barfin flounder nervous necrosis virus
RdRp: RNA dependent RNA polymerase
wt: wild type
M.O.I: multiplicity of infection
pi: post-infection
CPE: cytopathic effect
ts: temperature sensitivity
TMD: transmembrane domain
MLS: membrane localization signals
NCR: non coding region
